# Supplementary material for: Continuous Protein Supplementation Reduces Acute Exercise-Induced Stress Markers in Athletes Performing Marathon
Source: Nutrients. 2021 Aug 24;13(9):2929. doi: 10.3390/nu13092929 (PMC8472015; doi:10.3390/nu13092929)
Supplement: Supplementary file 1 [file nutrients-13-02929-s001.zip › nutrients-1328958-supplementary.pdf]

Supplementary Table S1. Energy (EI) and macronutrient intake (expressed as a percentage of EI and relative to BW) in the tested study groups (Verum, Control) at the beginning (time point a) and end (time point b) of the intervention.

**Variable**	**Verum (*n* = 10)**		**Control (*n* = 13)**	
	**a**	**b**	**a**	**b**
EI (kcal/d)	2550 ± 860	2610 ± 570	2127 ± 600	2192 ± 630
Protein (E%)	15 ± 2**	23 ± 3††	16 ± 4	16 ± 3
Fat (E%)	32 ± 6*	28 ± 4†	33 ± 6	33 ± 5
CHO (E%)	51 ± 5	50 ± 4	48 ± 7	51 ± 5
Protein (g/kg)	1.2 ± 0.4**	1.8 ± 0.2††	1.2 ± 0.4	1.2 ± 0.4
Fat (g/kg)	1.2 ± 0.4	1.0 ± 0.3	1.1 ± 0.3	1.1 ± 0.4
CHO (g/kg)	4.1 ± 1.3	4.4 ± 0.9	3.5 ± 0.9	3.7 ± 0.9

The results are presented as mean ± SD. *p<0.05 and **p<0.01 (intra-group comparisons between time points). †p<0.05 and ††p<0.01 (inter-group comparisons at time point).
